# Transcriptomic profiling reveals substrate- and shear stress-dependent maturation of human small intestinal epithelial cells

**DOI:** 10.3389/fphar.2026.1706106

**Published:** 2026-05-01

**Authors:** Nadra Alzain, Ahed Almalla, Ketaki Sandu, Lorenz Gerbeth, Jörn F. Ziegler, Catharina Gutwein, Patrick Weidner, Rainer Glauben, Marie Weinhart, Britta Siegmund

**Affiliations:** 1 Department of Gastroenterology, Infectious Diseases and Rheumatology, Charité - Universitätsmedizin Berlin, Corporate Member of Freie Universität Berlin and Humboldt-Universität zu Berlin, Berlin, Germany; 2 Institute of Chemistry and Biochemistry, Freie Universität Berlin, Berlin, Germany; 3 BIH Charité Junior Clinician Scientist Program, Berlin Institute of Health at Charité – Universitätsmedizin Berlin, Berlin, Germany; 4 Berlin Institute for Medical Systems Biology, Max Delbrück Center for Molecular Medicine in the Helmholtz Association, Berlin, Germany; 5 Berlin Institute of Health at Charité – Universitätsmedizin Berlin, Berlin, Germany; 6 Institute of Physical Chemistry and Electrochemistry, Leibniz Universität Hannover, Hannover, Germany; 7 Cluster of Excellence ImmunoPreCept, Charité – Universitätsmedizin Berlin, Berlin, Germany

**Keywords:** biomimetic, epithelial cells, hydrogel, millifluidics, organoids, shear stress, small intestine

## Abstract

Primary human intestinal epithelial cells (IECs) require microenvironments that reproduce various *in vivo* cues to maintain survival, differentiation, and function *in vitro*. In this study, we investigated how intestinal stem cell (ISC)-derived monolayers respond to biomimetic substrates and shear stress using 3D-printed hydrogels based on bioactive decellularized and methacrylated small intestinal submucosa (dSIS-MA) integrated into a custom millifluidic system. Extensive bulk RNA sequencing experiments revealed that, compared with Matrigel-coated tissue culture plastic, dSIS-MA hydrogels supported survival- and differentiation-related signaling, stabilized gene expression over time, and promoted absorptive lineage maturation while reducing crypt-associated signatures. By applying dynamic culture conditions to the hydrogel system, IECs underwent transcriptional remodeling, characterized by activation of metabolic and immune pathways. Longitudinal analysis further indicated that shear stress enhanced metabolic pathway–associated gene expression and promoted differentiation toward absorptive lineages. These findings establish dSIS-MA hydrogels with controlled fluid flow as a biomimetic *in vitro* model that supports survival, maturation of human IECs and enables transcriptional adaptation to defined biochemical and mechanical cues, supporting future applications in disease modelling, drug testing, and regenerative medicine.

## Introduction

The intestinal epithelium forms a dynamic barrier that mediates interactions between the host and its environment while enabling nutrient absorption. This function is supported by continuous epithelial renewal and the crypt–villus architecture, in which stem cells give rise to differentiated cell types (Turner, 2009; Schofield et al., 1993).

The small intestinal epithelium comprises absorptive and secretory lineages derived from Lgr5^+^ stem cells within the crypts. Differentiated cell types include enterocytes, goblet cells, Paneth cells, and enteroendocrine cells, which together support barrier function and intestinal homeostasis. Proliferation and differentiation of these cells are tightly regulated by multiple signaling pathways, including WNT, NOTCH, EGF, BMP, and Hippo (Beumer and Clevers, 2021; Takahashi and Shiraishi, 2020). The ECM forms a dynamic microenvironment that provides crucial biochemical and biomechanical signals for cell survival, proliferation, and differentiation (Mouw et al., 2014).

Traditional *in vitro* models using transformed cell lines, mostly colonic epithelial cells grown on rigid synthetic substrates, fail to replicate the complex, dynamic microenvironment of the intestine, lacking cellular diversity, ECM support, and physiological mechanical forces such as shear stress (Costa and Ahluwalia, 2019). Advances such as intestinal organoids derived from primary stem cells have improved physiological relevance by recapitulating cellular heterogeneity and differentiation capacity (Däullary et al., 2020; Sato et al., 2009). However, the enclosed 3D structure of organoids restricts access to the apical surface, limiting functional assays and physiological modeling. Two-dimensional organoid-derived monolayers overcome this limitation by providing access to both apical and basolateral compartments (Roodsant et al., 2020).

To further enhance physiological relevance, natural ECM-based hydrogels such as porcine small intestinal submucosa (SIS) have been introduced. Initial studies indicate that SIS mimics the biochemical composition and microarchitecture of native intestinal ECM, supporting cell adhesion, proliferation, and differentiation better than synthetic substrates (Cao et al., 2019; Zhao et al., 2024). Bioactive resins derived from SIS enable 3D printing of hydrogel scaffolds that replicate native intestinal tissue topology, offering precise control over microenvironmental features (Elomaa et al., 2023). However, a systematic analysis comparing culture on classic cell culture plates with culture on ECM-based hydrogels is lacking.

Mechanical forces, particularly shear stress from peristalsis and luminal flow, are critical regulators of intestinal epithelial physiology. Mechanotransduction pathways enable cells to convert shear stress into biochemical signals, influencing cell behavior and adhesion dynamics (Dawson et al., 2023; Espina et al., 2023). The gut-on-a-chip platform pioneered by Kim et al. integrates fluid flow and mechanical strain to simulate peristalsis and shear stress, advancing the study of epithelial responses under physiologically relevant conditions (Kim and Ingber, 2013). Millifluidic systems offer a more accessible alternative with flexibility in substrate choice and ease of use, though most studies remain limited to cell lines, with organoid integration emerging more recently (Alzheimer et al., 2020; Fois et al., 2021; Kim et al., 2012).

Building on these advances, next-generation intestinal models increasingly integrate organoid-derived epithelial cells with biomimetic hydrogels and dynamic culture systems to better emulate the complex *in vivo* intestinal environment, providing more accurate control over mechanical and biochemical signals (Däullary et al., 2020). Our previously developed hydrogel-integrated millifluidic platform, which utilizes 3D-printed decellularized small intestinal submucosa methacrylate (dSIS-MA) hydrogels seeded with human ileal intestinal stem cells, enables the application of physiological shear stress and supports spontaneous differentiation without the need for exogenous factors (Almalla et al., 2024).

Here we test whether a hydrogel-integrated millifluidic platform combining organoid-derived human ileal epithelial monolayers, a native ECM-based hydrogel, and controlled shear stress can sustain and guide their maturation and functional differentiation. Using bulk RNA sequencing at defined time points, we chart survival, proliferation, and differentiation programs, establishing a resource for studying intestinal homeostasis and disease.

## Materials and methods

### Organoid culture

Human intestinal organoids were established from terminal ileum biopsies of two healthy female donors, with approval from the ethics committee of Charité–Universitätsmedizin Berlin and written informed consent was obtained from all donors prior to the study. Organoids were generated and maintained as described previously (Almalla et al., 2024; Sato et al., 2011). Briefly, crypts were isolated by incubation in chelation buffer and embedded in growth factor–reduced Matrigel domes. Organoids were cultured in organoid expansion medium consisting of WRN-conditioned medium produced from L-WRN cells (ATCC CRL-3276), supplemented with recombinant human EGF, SB202190, A83-01, Gastrin I, Nicotinamide, and Y-27632 during initial passages. Antibiotics were included during establishment to prevent contamination. Cultures were maintained at 37 °C and 5% CO_2_, with medium exchange every other day and passaged weekly. Organoids were passaged weekly (7–12 days) at an approximate split ratio of 1:6. For passaging, Matrigel domes were mechanically disrupted and organoids collected in basal medium, followed by centrifugation. Organoids were then enzymatically dissociated using TrypLE™ Express supplemented with Y-27632 at 37 °C, with gentle mechanical trituration to obtain small clusters or single cells as required. Cells were washed, resuspended in Matrigel, and reseeded. To maintain culture quality, organoids were routinely assessed based on morphology, and only cystic, undifferentiated structures were selected for passaging. Organoids at passages five to six were used for subsequent experiments.

### Shear stress parameters and flow settings

Shear stress within the dynamic tissue culture chambers was applied using a peristaltic pump maintaining a constant flow rate of 1.42 mL/min through a laminar flow channel with dimensions length 2.4 cm, width 2.0 cm, and height 2.5 mm. The resulting surface shear stress on the cellularized hydrogels was estimated based on the channel geometry, medium viscosity (0.93 mPa s), and volumetric flow rate. Computational fluid dynamics simulations (COMSOL v5.5) were used to model velocity fields and a physiological shear stress was applied constantly throughout the experiment. To reproduce the physiological shear stress on small intestinal cells with regard to interdigestive states, lower shear levels on the villi bottom and beneath the mucus layer, commonly applied shear stress levels from 0.02 were reduced to 0.01 dyne/cm^2^.

For dynamic culture, the perfusion circuit (excluding the pump) was packaged in Steriking® sterilization pouches, sealed, and autoclaved at 120 °C. Medium exchange (12 mL) was performed every 7 days.

### Hydrogel preparation, sterilization and seeding of monolayers

Decellularized and methacrylated porcine small intestinal submucosa (dSIS-MA) was processed into flat 3D-printed hydrogel discs (6 mm × 1 mm) as described previously (Almalla et al., 2024). Detailed physicochemical and mechanical characterization of the dSIS-MA hydrogels included nuclear magnetic resonance spectroscopy, circular dichroism, rheology, atomic force microscopy, scanning electron microscopy, and chromogenic assays.

For cell culture, hydrogels were disinfected in 70% ethanol 1 h, followed by three washes in sterile PBS (−/−) containing 1× penicillin/streptomycin, each lasting 1 h. Disinfected hydrogels were stored in sterile PBS (−/−) at 4 °C in 96-well plates until cell seeding where they were pre-equilibrated in culture medium (Almalla et al., 2024).

Organoids were dissociated into single cells using TrypLE Express and gentle mechanical trituration. Cells were seeded in organoid expansion medium supplemented with Y-27632 at 1 × 10^5^ cells per hydrogel (∼3.54 × 10^5^ cells/cm^2^), with tissue culture plastic (TCP) controls seeded at a comparable density on Matrigel-coated plastic in the same plate, normalizing for the well surface area. Following sedimentation and attachment, monolayers were cultured for 2 days before medium replacement.

Custom-designed biocompatible dynamic culture chambers were produced by 3D printing and assembled under sterile conditions as described previously (Almalla et al., 2024). Confluent monolayer-bearing hydrogels were transferred into the chambers, sealed with gaskets, and connected via tubing to a closed-loop perfusion circuit with a medium reservoir and 0.22 µm filter. Flow was driven by an Arduino-controlled pump located outside the incubator, while the chamber and reservoir were maintained at 37 °C and 5% CO_2_. After experiments, hydrogels were retrieved for downstream analyses.

### RNA isolation, sequencing, and analysis

Experiments were conducted using two biological replicates and three to four technical replicates per condition. Hydrogels were lysed in QIAzol reagent and RNA extracted using the Direct-zol RNA Microprep kit (Zymo Research). RNA integrity was assessed on an Agilent TapeStation (RNA ScreenTape assay, Software A.02.01 SR1), and samples with RNA integrity number (RIN) ≥ 8 were used for downstream analyses. Bulk RNA sequencing and analysis was performed by Novogene (Munich). Libraries were prepared using the Illumina mRNA library preparation kit with poly-A enrichment from the recommended Novogene RNA input. Sequencing was performed on an Illumina platform using paired-end 150 bp reads, generating approximately 9 Gb of data per sample (∼30 million reads per sample). Paired-end reads were aligned to the human reference genome (GRCh38, GENCODE v33) using HISAT2 v2.0.5 with default parameters. Gene-level counts were obtained using featureCounts v1.5.0-p3. Differential expression analysis was performed using DESeq2 v1.20.0 for samples with biological replicates, and edgeR v3.22.5 for samples without replicates, using a |log_2_(FoldChange)| ≥ 1 and adjusted p-value ≤0.05 as significance thresholds. P-values were corrected for multiple testing using the Benjamini–Hochberg method. Data visualization was carried out using R v4.4.2.

### cDNA synthesis and quantitative PCR

For validation, RNA was reverse-transcribed using M-MLV reverse transcriptase with Oligo (dT) primers. qPCR was performed with SYBR Green Master Mix on a QuantStudio 5 Real-Time PCR System (Applied Biosystems). Gene expression was normalized to TBP, POLR2A, and HPRT reference genes. Primer sequences are provided in Supplementary Table S1. RT-qPCR analyses were performed to technically validate RNA-seq expression trends and were not intended to provide additional biological replication.

### Reagents and chemicals

All reagents and chemicals are summarized in Supplementary Table S2.

## Results

**FIGURE 1:**
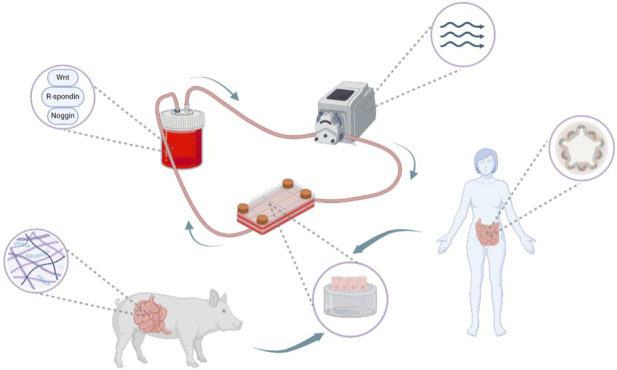
Hydrogel-integrated millifluidic system for intestinal modeling. Schematic of the millifluidic setup featuring porcine small intestinal-derived hydrogel scaffolds for human primary small intestinal epithelial cells. The system includes a tissue chamber for cells and hydrogels, a media reservoir containing expansion media, and a peristaltic pump to induce fluid flow. Figure created with BioRender.com.

To evaluate how primary human ileal intestinal epithelial cells (IECs) respond to physiologically relevant cues, we cultured intestinal stem cell (ISC)-derived monolayers on either Matrigel-coated tissue culture plastic (TCP) or 3D-printed dSIS-MA hydrogels, under static or dynamic flow in a custom millifluidic system (Figure 1) (Almalla et al., 2024). Bulk RNA sequencing was performed to assess transcriptional responses to substrate composition and shear stress.

### Hydrogel substrates promote enhanced IEC signaling

**FIGURE 2:**
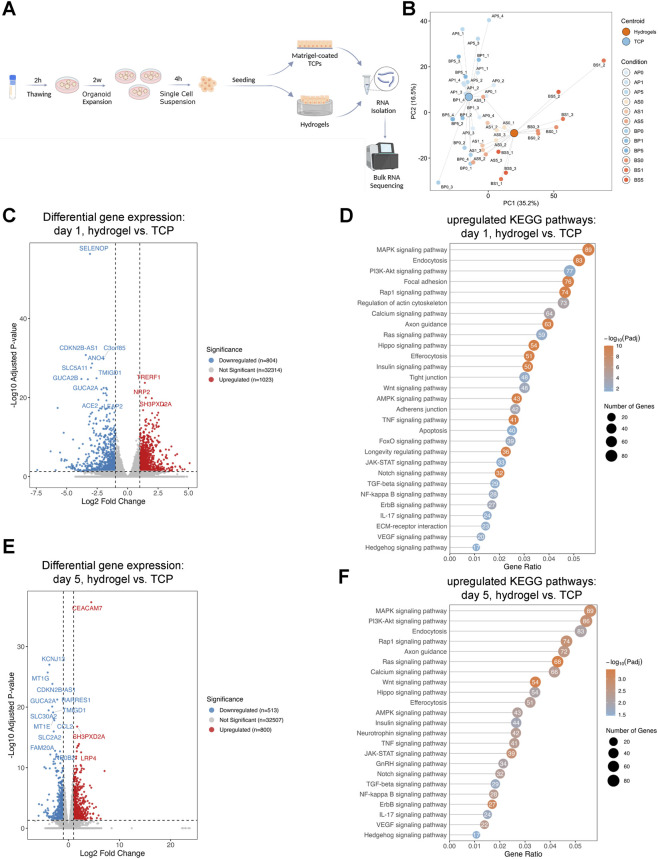
Upregulation of survival, proliferation, and differentiation signaling pathways in intestinal stem cell-derived monolayers cultured on hydrogels compared to tissue culture plastic. **(A)** Experimental set-up: small intestinal organoids were expanded, dissociated into single cells, and seeded on hydrogel substrates or Matrigel-coated tissue culture plastic (TCP). Samples were collected at days 0, 1 and 5 post-confluency for RNA extraction and bulk RNA-seq. Figure created with BioRender.com. **(B)** Principal component analysis (PCA) plot of gene expression profiles from cultures on hydrogels (orange) and TCP (blue) across time points. Large dots mark condition centroids; small dots represent technical replicates labeled by donor, substrate, day, and replicate (A/B = donor; P/S = plastic/scaffold; 0/1/5 = day; 1–4 = technical replicate). The separation of clusters across principal components highlights consistent and reproducible gene expression differences between the two culture substrates and across time points. **(C)** Volcano plot depicting differentially expressed genes between cells cultured on hydrogels vs. TCP on day 1. The x-axis represents the log_2_ fold change in gene expression, while the y-axis shows the −log_10_ of the adjusted p-value, indicating statistical significance. Genes with adjusted p-value ≤0.05 and absolute log_2_ fold change ≥1 are highlighted (red = upregulated, blue = downregulated, grey = not significant). **(D)** KEGG pathway enrichment analysis (upregulated genes) in intestinal stem cell-derived monolayers cultured on hydrogels compared to TCP on day 1. Bubble size indicates the number of genes per pathway, color denotes–log_10_ adjusted p-value, and the x-axis shows the gene ratio (upregulated genes per pathway/total genes in pathway). **(E)** Volcano plot showing differentially expressed genes between intestinal stem cell-derived monolayers cultured on hydrogels and TCP on day 5, analyzed as in panel **(C)**. **(F)** KEGG pathway enrichment analysis of upregulated genes in intestinal stem cell-derived monolayers cultured on hydrogels vs. TCP at day 5, analyzed as in panel **(D)**.

Ileal ISC-derived monolayers were prepared as outlined in Figure 2A and seeded onto either Matrigel-coated TCP or dSIS-MA hydrogels. The time point at which monolayers reached confluency was designated as day 0. Cells were subsequently harvested, processed, and RNA-seq analysis was performed at two time points: day 1 and day 5 post-confluency.

RNA-seq analysis revealed distinct transcriptional profiles between ISC-derived monolayers cultured on dSIS-MA hydrogels and those grown on Matrigel-coated TCP under static conditions across both day 1 and day 5. Principal component analysis (PCA) demonstrated clear separation between the groups, with PC1 (35.2%) and PC2 (16.5%) together accounting for 51.7% of the variance (Figure 2B). At day 1, a total of 1,023 genes were up- and 804 downregulated in cells grown on dSIS-MA hydrogels compared to Matrigel-coated TCP (Figure 2C). KEGG pathway enrichment revealed significant higher activation of key pathways associated with cell survival, proliferation, and differentiation, including MAPK, Rap1, PI3K-Akt, focal adhesion, and endocytosis (Figure 2D). By day 5, a total of 800 genes were upregulated and 513 downregulated (Figure 2E). KEGG pathway enrichment revealed sustained activation of similar pathways such as MAPK, focal adhesion, Rap1, and calcium signaling, as well as additional pathways including autophagy, insulin, and TGF-β signaling (Figure 2F). These results indicate that dSIS-MA hydrogels promote sustained activation of pro-survival and differentiation-related signaling in ISC-derived monolayers.

### Sustained gene expression in dSIS-MA hydrogels and declining survival pathways on Matrigel-coated TCP

**FIGURE 3:**
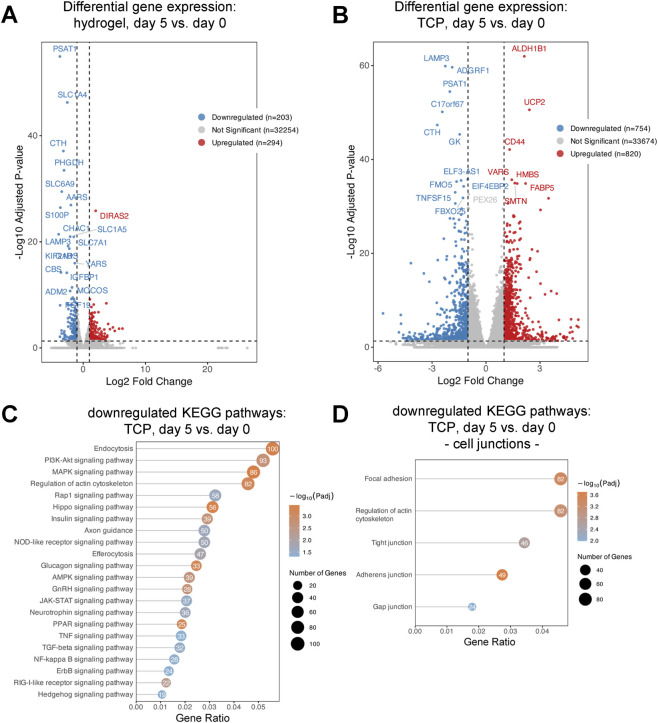
Intestinal stem cell-derived monolayers exhibit stable gene expression on hydrogels, but downregulate survival pathways on tissue culture plastic over time. Volcano plot shows differentially expressed genes between day 0 and day 5 for **(A)** cells cultured on hydrogels and **(B)** cells cultured on tissue culture plastic (TCP). The x-axis represents the log_2_ fold change in gene expression, while the y-axis depicts the −log_10_ of the adjusted p-value, indicating statistical significance. Genes with adjusted p-value ≤0.05 and absolute log_2_ fold change ≥1 are highlighted (red = upregulated, blue = downregulated, grey = not significant). These plots reveal minimal transcriptional changes over time on hydrogels compared to substantial alterations in gene expression on TCP. **(C, D)** KEGG enrichment bubble plot showing the most significantly downregulated pathways in monolayers on TCP from day 0 to day 5. The x-axis indicates the gene ratio, the y-axis lists KEGG pathways, bubble size reflects gene count, and color denotes–log_10_ adjusted p-value.

To assess the temporal stability of gene expression across different culture systems, a comparative analysis of gene expression profiles was performed between day 0 and day 5. This analysis demonstrated that cell cultures on dSIS-MA hydrogels maintained relatively stable gene expression profiles over time, as evidenced by the upregulation of 294 genes without the emergence of any enriched KEGG pathways, suggesting minimal transcriptional alterations during the culture period (Figure 3A). Conversely, cells maintained as monolayers on Matrigel-coated TCP exhibited a substantial downregulation of 754 genes over the same period (Figure 3B). Subsequent KEGG pathway analysis of the downregulated genes in Matrigel-coated TCP monolayers revealed decreased expression in several critical signaling pathways associated with cell survival and growth, including TNF, TGF-β, Rap1, AMPK, PI3K-Akt, and MAPK signaling pathways (Figure 3C). Additionally, pathways fundamental to intercellular communication and structural integrity, such as focal adhesion, regulation of the actin cytoskeleton, adherens junctions, tight junctions, and gap junctions, were also significantly downregulated (Figure 3D). In contrast, these same pathways remained upregulated in dSIS-MA hydrogel-cultured monolayers at both time points, highlighting a marked divergence in the temporal regulation of gene expression and cellular signaling dynamics between the two culture systems.

### dSIS-MA hydrogels promote differentiation of intestinal stem cell-derived monolayers

To assess the differentiation status of ISC-derived monolayers cultured on dSIS-MA hydrogels vs. Matrigel-coated TCP, we referenced the cell type-specific gene signatures defined by Burclaff et al., which defined small intestine-specific markers across three epithelial lineages (Burclaff et al., 2022). The absorptive lineage comprised early, intermediate, and mature absorptive enterocytes (AE), with AE2 clustering separately, as well as bestrophin 4 (BEST4)-expressing cells. The secretory lineage included follicle-associated epithelium (FAE), tuft cells, enteroendocrine cells (EEC), and goblet cells. The crypt-based lineage included transit amplifying cells (TA, TA2), intestinal stem cells (ISC), secretory progenitors, and Paneth cells.

**FIGURE 4:**
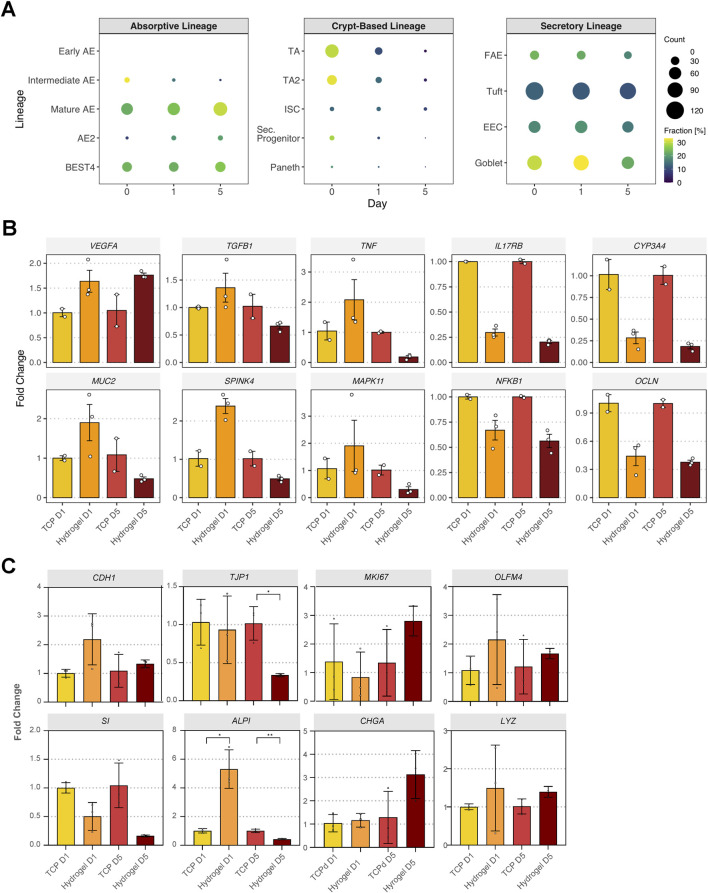
Extracellular matrix hydrogels promote differentiation of intestinal stem cell-derived monolayers. **(A)** Dot plots of upregulated genes in absorptive, secretory, and crypt-based lineages of cells cultured on hydrogels vs. tissue culture plastic across days 0, 1, and 5. Bubble size reflects gene count, and color indicates the fraction (%) of genes expressed per cell type. Absorptive lineage: Early AE (absorptive enterocyte), Intermediate AE, Mature AE, AE2 (subtype 2), BEST4 (bestrophin-4 expressing AE); Crypt-based lineage: TA (transit-amplifying cell), TA2, ISC (intestinal stem cell), Sec. progenitor (secretory progenitor cell), Paneth; Secretory lineage: FAE (follicle-associated epithelial cell), Tuft, EEC (enteroendocrine cell), Goblet. **(B, C)** Relative expression analysis of selected genes in monolayers cultured on tissue culture plastic (TCP) or hydrogels for 1 day (D1) or 5 days (D5) (two (B) or three **(C)** technical replicates for TCP and three for hydrogels). Data were normalized to TCP at the corresponding time point and are shown as mean ± SD. Unpaired t-test with Welch’s correction was performed, *p < 0.05, **p < 0.01.

When comparing dSIS-MA hydrogels vs. Matrigel-coated TCP, gene expression analysis of ISC-derived monolayers showed no early AE marker expression, a decline in intermediate AE markers, and slight increases in mature AE, AE2, and BEST4 markers over time. Secretory lineage expression decreased by day 5, though goblet cell genes remained stable between days 0 and 1. Crypt-based lineage markers declined across time points (Figure 4A).

To validate the RNA-seq results, RT-qPCR was performed using one biological replicate. Consistent with the RNA-seq data, *VEGFA*, *TGFβ1*, and *TNF* were upregulated on hydrogels on day 1, while *IL17RB* and *CYP3A4* were downregulated compared to those on TCP. Expression of *MAPK11*, *MUC2*, and *SPINK4* decreased on day 5. However, some discrepancies were observed: *MUC2* and *SPINK4* were upregulated on day 1 by RT-qPCR, contrary to RNA-seq findings. *NFKB1* and *OCLN* were reduced in RT-qPCR, whereas RNA-seq showed no change for *NFKB1* and a decrease in *OCLN* only on day 1. Additionally, *MAPK11* expression increased on day 1 in RT-qPCR but not in RNA-seq data (Figure 4B).

To further assess epithelial maturation and lineage specification, additional differentiation markers were analyzed by RT-qPCR using cDNA from an independent experiment (Figure 4C). On dSIS-MA hydrogels, early increases in *CDH1* and *ALPI* expression were observed at day 1 compared with TCP, consistent with the enrichment of adherens junction and differentiation-associated pathways identified by RNA-seq. At later time points, *MKI67* expression remained elevated on hydrogels, suggesting sustained proliferative potential, while *CHGA* and *LYZ* expression indicated maintenance of secretory and enteroendocrine lineage-associated transcriptional programs. *OLFM4* expression showed modest early enrichment on hydrogels, in line with sustained activation of proliferation-associated pathways. Although individual gene trends varied, the overall marker profile supports scaffold-associated stabilization of survival, proliferation, and differentiation-related transcriptional signatures observed in the pathway-level analyses.

Overall, ISC-derived monolayers on dSIS-MA hydrogels showed a trend toward absorptive lineage maturation with reduced secretory and crypt-based signatures, though some discrepancies between RNA-seq and RT-qPCR warrant cautious interpretation.

### Shear stress induces distinct gene expression on dSIS-MA hydrogels

**FIGURE 5:**
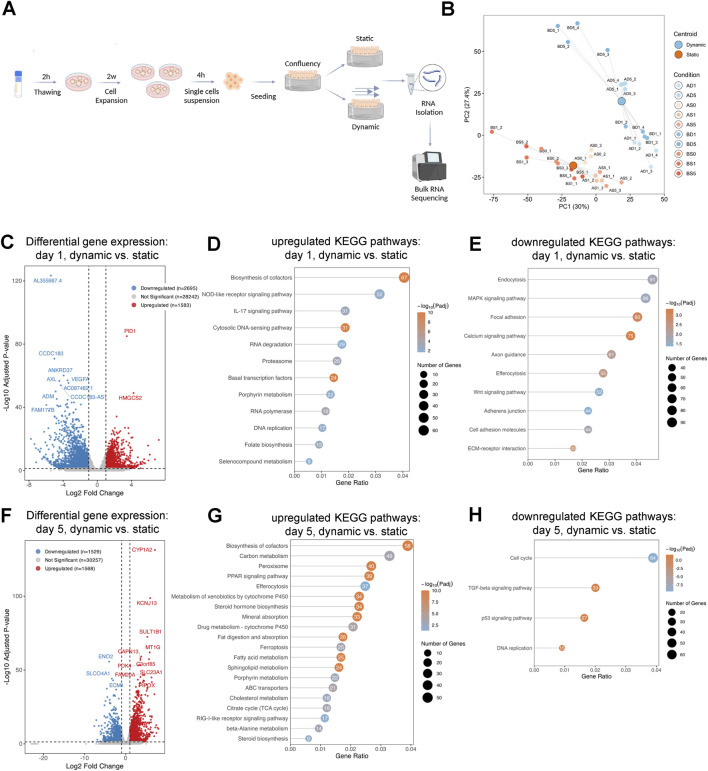
Shear stress induces distinct gene expression in intestinal stem cell-derived monolayers on hydrogels. **(A)** Experimental set-up: organoids were expanded and subsequently dissociated to generate epithelial monolayers, seeded on hydrogels, and cultured under static or dynamic (shear stress) conditions for 0, 1 or 5 days before RNA-seq. Figure created with BioRender.com. **(B)** Principal component analysis (PCA) plot showing distinct expression profiles between static and dynamic conditions. Sample labels indicate donor **(A, B)**, culture condition (S = static, D = dynamic), day (0, 1, 5), and replicate (Turner, 2009; Schofield et al., 1993; Beumer and Clevers, 2021; Takahashi and Shiraishi, 2020). The separation of clusters across principal components highlights consistent and reproducible gene expression differences between the static and dynamic conditions. **(C)** Volcano plot showing the differentially expressed genes in dynamic vs. static conditions on day 1. The x-axis represents the log_2_ fold change in gene expression, while the y-axis shows the -log_10_ of the adjusted p-value, indicating statistical significance. Genes with adjusted p-value ≤0.05 and absolute log_2_ fold change ≥1 are highlighted (red = upregulated, blue = downregulated, grey = not significant). Bubble charts depict the most significantly **(D)** upregulated and **(E)** downregulated KEGG pathways in intestinal stem cell-derived monolayers cultured under dynamic vs. static conditions on day 1. The x-axis represents the gene ratio, the y-axis the pathways, bubble size reflects gene count, and color denotes–log_10_ adjusted p-value. **(F)** Volcano plot showing the differentially expressed genes in dynamic vs. static conditions on day 5, analyzed as in panel **(C)**. **(G)** upregulated and **(H)** downregulated KEGG pathways under dynamic vs. static conditions on day 5, analyzed as in panel **(D, E)**.

Given the clear advantages observed in cell survival, proliferation, and differentiation of small intestinal IEC monolayers cultured on dSIS-MA hydrogels, subsequent experiments were conducted exclusively under this condition (Figure 5A). PCA (Figure 5B) revealed distinct clustering of ISC-derived monolayers cultured on dSIS-MA hydrogels under dynamic vs. static conditions, with PC1 (30%) and PC2 (27.4%) accounting for 57.4% of the variance. At 24 h, biological replicates under dynamic conditions clustered tightly, whereas slight separation emerged by day 5. In contrast, under static conditions, biological replicate A showed consistent clustering over time, while replicate B exhibited increased dispersion.

On day 1, dynamic culture resulted in higher expression of 1,583 genes and lower expression of 2,695 genes relative to static conditions (Figure 5C). KEGG enrichment analysis revealed pathways with higher expression, including biosynthesis of cofactors, NOD-like receptor signaling, IL-17 signaling, cytosolic DNA sensing, and porphyrin metabolism (Figure 5D). Conversely, pathways with lower expression encompassed calcium signaling, focal adhesion, ECM-receptor interaction, MAPK signaling, Wnt signaling, and adherens junctions (Figure 5E).

By day 5, a total of 1,568 genes were expressed at higher levels and 1,529 at lower levels under dynamic conditions compared to static conditions (Figure 5F). Upregulated pathways included PPAR signaling, ferroptosis, porphyrin metabolism, cytochrome P450-mediated drug metabolism, and RIG-I-like receptor signaling (Figure 5G). Key downregulated pathways comprised TGF-β signaling, p53 signaling, and cell cycle regulation (Figure 5H).

Together, these results demonstrate that dynamic culture on dSIS-MA hydrogels elicits a robust and time-dependent transcriptional response, characterized by the activation of metabolic and immune-related pathways and the suppression of proliferation- and cell cycle–associated programs.

### Temporal gene expression and pathway shift on dSIS-MA hydrogels under static vs. dynamic conditions

**FIGURE 6:**
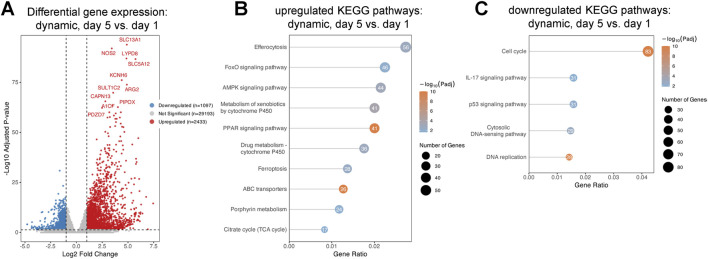
Temporal differential gene expression and pathway enrichment in intestinal stem cell-derived monolayers under dynamic conditions. **(A)** Volcano plot showing the differentially expressed genes under dynamic compared to static conditions over time (day 5 vs. day 1). The x-axis represents the log_2_ fold change in gene expression, while the y-axis shows the −log_10_ of the adjusted p-value, indicating statistical significance. Genes with adjusted p-value ≤0.05 and absolute log_2_ fold change ≥1 are highlighted (red = upregulated, blue = downregulated, grey = not significant). KEGG enrichment bubble plot showing the most significantly **(B)** upregulated and **(C)** downregulated pathways in monolayers under dynamic vs. static conditions over time (day 5 vs. day 1). The x-axis indicates the gene ratio, the y-axis lists KEGG pathways, bubble size reflects gene count, and color denotes–log_10_ adjusted p-value.

Differential gene expression analysis over time (day 1 to day 5), comparing ISC-derived monolayers cultured on dSIS-MA hydrogels under dynamic vs. static conditions, revealed 2,433 upregulated and 1,097 downregulated genes (Figure 6A). KEGG pathway enrichment analysis indicated upregulation of pathways including ferroptosis, porphyrin metabolism, efferocytosis, PPAR signaling, AMPK, FOXO, and cytochrome P450 drug metabolism (Figure 6B). Additional upregulated pathways included those related to lipid, cholesterol, sugar, amino acid, and fatty acid metabolism, as well as nutrient absorption and biosynthesis. Downregulated pathways involved IL-17 and p53 signaling, cytosolic DNA sensing, cell cycle, and DNA replication (Figure 6C).

These findings suggest that shear stress promotes metabolic reprogramming and transcriptional features consistent with epithelial maturation of ISC-derived monolayers while downregulating inflammatory and proliferative signaling pathways over time.

### Shear stress enhances differentiation of intestinal stem cell-derived monolayers

**FIGURE 7:**
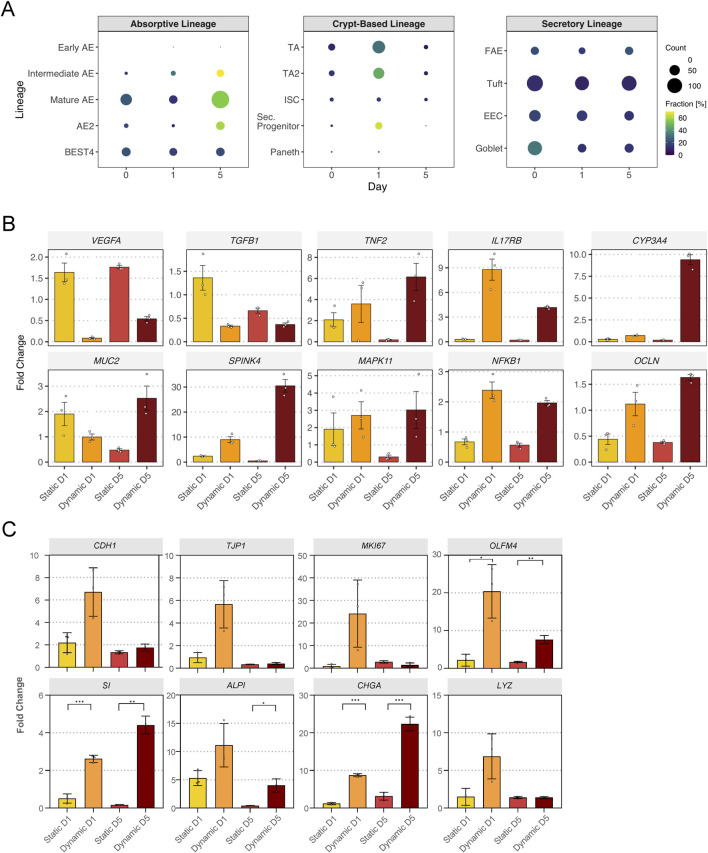
Shear stress enhances the cell differentiation of intestinal stem cell-derived monolayers. **(A)** Dot plots of upregulated genes in absorptive, secretory, and crypt-based lineages of cells cultured under dynamic vs. static conditions across days 0, 1, and 5. Bubble size reflects gene count, and color indicates the fraction (%) of genes expressed per cell type. Absorptive lineage: Early AE (absorptive enterocyte), Intermediate AE, Mature AE, AE2 (subtype 2), BEST4 (bestrophin-4 expressing AE); Crypt-based lineage: TA (transit-amplifying cell), TA2, ISC (intestinal stem cell), Sec. progenitor (secretory progenitor cell), Paneth; Secretory lineage: FAE (follicle-associated epithelial cell), Tuft, EEC (enteroendocrine cell), Goblet. **(B, C)** Relative expression analysis of selected genes in monolayers cultured under dynamic vs. static conditions for 1 day (D1) or 5 days (D5). Data were normalized to TCP at the corresponding time point and are shown as mean ± SD (three technical replicates for TCP and hydrogels). Unpaired t-test with Welch’s correction was performed, *p < 0.05, **p < 0.01, ***p < 0.001.

To evaluate the differentiation of ISC-derived monolayers cultured on dSIS-MA hydrogels under dynamic vs. static conditions, we analyzed ileal-specific markers as defined by Burclaff et al. (2022). Absorptive lineage genes–including intermediate AE, mature AE, and AE2 – were strongly upregulated on day 5, with a moderate increase in early AE markers. BEST4 expression remained unchanged. Secretory lineage genes remained stable overall, although goblet cell markers declined on both days 1 and 5. Within crypt-based lineage, TA, TA2, and secretory progenitor genes increased on day 1, whereas Paneth cell and ISC markers remained rather stable (Figure 7A). An independent RT-qPCR experiment validated these findings by analyzing genes consistently identified in RNA-seq data. *TNF*, *IL17RB*, *SPINK4*, *MAPK11*, and *NFΚB1* were upregulated under dynamic conditions on both days 1 and 5. *CYP3A4* and *MUC2* expression increased on day 5, while *VEGFA* and *TGFβ1* were downregulated at both time points. *OCLN* was upregulated in RT-qPCR, but remained unchanged in RNA-seq, representing the sole notable discrepancy (Figure 7B). Under dynamic culture conditions, additional RT-qPCR analysis revealed time-dependent transcriptional changes consistent with the RNA-seq pathway enrichment data (Figure 7C). At day 1, increased expression of *CDH1*, *TJP1*, *MKI67*, and *OLFM4* was observed under dynamic conditions compared with static culture, suggesting transient enhancement of junction-associated and proliferative gene expression. By day 5, elevated expression of *SI*, *ALPI*, and *CHGA* under dynamic conditions indicated enhanced absorptive and enteroendocrine lineage-associated transcriptional programs. These gene-level observations align with KEGG analyses demonstrating an early proliferative signature followed by enrichment of metabolic and maturation-related pathways under shear stress.

Overall, these results indicate that culturing small intestinal epithelial monolayers on dSIS-MA hydrogels under dynamic conditions promotes differentiation toward absorptive lineage, supporting transcriptional maturation of ISC-derived monolayers in a physiologically relevant microenvironment.

## Discussion

In this study, we show that defined combinations of extracellular matrix and mechanical stimulation induce distinct and time-dependent transcriptional responses in human intestinal epithelial cells (IECs). We therefore present a transcriptomic analysis of primary human IECs cultured in a biomimetic microenvironment that integrates extracellular matrix features with controlled mechanical stimulation. Traditional cell culture models, which often rely on synthetic substrates and immortalized cell lines, fall short in replicating the complex architecture and function of the native intestinal epithelium.

### Integration of native ECM and shear stress in intestinal models

Recent gut-on-chip and organoid-on-chip models have successfully reproduced key aspects of intestinal physiology and disease, including cytokine-induced barrier disruption and inflammatory crosstalk with immune and stromal cells (Amatya et al., 2017; Du et al., 2023). Many of these systems are based on polydimethylsiloxane (PDMS) microfluidic chips with flat or porous membranes and standard ECM coatings such as collagen or Matrigel, where physiological relevance is mainly introduced through dynamic cues such as shear flow and cyclic peristalsis-like stretch, while the underlying substrate remains synthetic (Du et al., 2023; Brian et al., 1990; Kim et al. 2016; Thummel et al., 1996).

In contrast, our platform combines patient-derived intestinal stem cell monolayers with a 3D printed dSIS-MA hydrogel scaffold in a millifluidic format, enabling the simultaneous application of shear stress and native, tissue-derived extracellular matrix features. Unlike membrane-based PDMS chips that rely on surface coatings, the dSIS-MA scaffold directly experiences fluid flow and provides biochemical features of the intestinal submucosa. This approach is consistent with emerging work using decellularized intestinal matrix hydrogels–dSIS-norbornene (NB) (Meran et al., 2020; Nie et al., 2006) to support polarized epithelial monolayers and barrier function under flow.

### Hydrogel-dependent effects on epithelial gene expression

Using ISC-derived monolayers from the human terminal ileum, we show that dSIS-MA hydrogels alone significantly enhance monolayer viability and epithelial maturation–associated gene expression, with higher expression of genes involved in cell survival, proliferation, differentiation, and junctional signaling associated with epithelial barrier organization. This platform represents an advanced biomimetic culture system which supports potential applications in *in vitro* disease modeling using patient-derived epithelial cells and preclinical drug testing, particularly in assessing epithelial differentiation and barrier-related responses under defined conditions, as well as regenerative medicine.

The gene expression analysis of ISC-derived monolayers cultured on dSIS-MA hydrogels vs. Matrigel-coated TCP showed a notably higher expression of multiple survival, proliferation, and differentiation pathways (e.g., MAPK, RAS, ERBB, PI3K/AKT), which are highly interconnected (Shin et al., 2020; Verhulsel et al., 2021; Yokoi et al., 2025). Key pathways associated with cell-cell and cell-ECM interactions, including the actin cytoskeleton, tight junctions, adherens junctions, focal adhesions, and ECM-receptor interactions, were also upregulated. These pathways are known to be involved in mechanosensing and activating the TGF-β pathway (Ballerini et al., 2025; Vera et al., 2024), underscoring the biomimetic advantage of the natural scaffold. Additional developmental and metabolic pathways, including WNT, Hedgehog, NOTCH, and AMPK signaling, as well as axon guidance, were upregulated, supporting enhanced differentiation and metabolic homeostasis on hydrogels (Cargnello and Roux, 2011; Gkantzou et al., 2026; Hynes and MacDonald, 2009; Luong et al., 2025; Widmann et al., 1999). Importantly, these pathways were further reinforced over time on hydrogels, but downregulated on Matrigel-coated TCP, emphasizing the biomimetic advantage of the dSIS-MA matrix.

Regarding cell differentiation, monolayers cultured on dSIS-MA hydrogels exhibited an early upregulation of goblet cell markers, with an increase in mature enterocyte markers and a decline in crypt-based markers over time. This indicates accelerated lineage transcriptional progression toward differentiated lineages driven by the hydrogel substrate, consistent with our previous protein-level findings (Elomaa et al., 2023).

Our dataset of primary human small intestinal monolayers is unique and extends transcriptomic knowledge beyond previous work in other cell types and matrices. Notably, one bulk RNA-seq study of HUVEC cells on SIS reported upregulation of Hippo, MAPK, and WNT signaling, but downregulation of focal adhesion and axon guidance (Yap et al., 2018). However, the pathways identified as downregulated were instead upregulated in our data, likely reflecting cell–type–specific biology.

### Shear stress–dependent transcriptional responses

We further subjected the monolayers cultured on dSIS-MA hydrogels to physiological shear stress generated by laminar fluid flow inside a custom-designed dynamic chamber. Under dynamic conditions, we observed an upregulation of innate immune response-associated pathways, including NOD-like receptor signaling, RIG-I-like receptor signaling, and DNA-sensing pathways, which sense cellular stress and damage, and activate the immune response to promote tissue repair (Evers et al., 2021; Laws and Bashaw, 2022; Singh et al., 2022). IL-17 signaling was also upregulated, contributing to immune activation via MAPK and NF-κB (Briscoe and Therond, 2013). These findings align with existing research, which demonstrates that mechanotransduction influences immune responses (Zhou et al., 2022). However, at later time points (day 5), IL-17 and DNA-sensing pathways were downregulated, suggesting adaptive dampening of inflammatory responses. A key finding was the shear stress–induced upregulation of CYP3A4, validated by qPCR, which was absent in static culture. This finding is relevant for future drug testing applications, as CYP3A4 is a well-established transcriptional marker of enterocyte maturation and metabolic potential (Herzig and Shaw, 2018; Yang et al., 2022). Our observation is consistent with prior research using mechanical stimulation of intestinal cells (Yu and Liu, 2021).

Pathways related to biosynthesis and metabolism (cofactors, sugars, amino acids, fatty acids, vitamins, minerals) were also enriched under shear stress, suggesting enhanced metabolic activity. On day 5, under dynamic conditions, a substantial upregulation in enterocyte gene expression was evident, which aligns with our published immunostaining data (Almalla et al., 2024). However, changes in secretory cell gene expression were less pronounced, although immunostaining data revealed an upregulation of goblet cell markers, reflecting possible divergence between transcriptional and protein-level regulation (Yu and Liu, 2021). Day 1 data showed an interesting enrichment in crypt-based cell lineage, indicating transient stimulation of the stem cell niche after acute exposure to shear stress.

The inclusion of additional lineage and maturation markers further supports the transcriptional trends identified by bulk RNA-seq. Gene-level analyses demonstrated early scaffold-associated upregulation of adherens junction and absorptive markers, sustained proliferative signatures at later stages, and maintenance of secretory lineage-associated transcriptional programs. Under dynamic conditions, early increases in proliferation- and junction-associated markers were followed by enhanced expression of absorptive and enteroendocrine differentiation markers. While these observations are based on limited biological replication and reflect transcriptional trends rather than functional validation, they provide additional support for scaffold- and shear-dependent modulation of epithelial differentiation programs.

### Implications for biomimetic intestinal modeling

Incorporating dSIS-MA hydrogels with human small intestinal organoid-derived monolayers enables a culture environment that more closely reflects key biochemical and mechanical features of the native intestinal epithelium. Compared with more mechanically complex gut-on-chip devices that integrate cyclic peristaltic stretch or multi-compartment co-cultures (Brian et al., 1990; Kim et al., 2016), our system uses a more streamlined mechanical design. Its main advantage is the combination of a native decellularized intestinal ECM scaffold with primary human ileal stem cell-derived monolayers under defined shear stress, allowing studies of epithelial differentiation and matrix-dependent transcriptional responses under flow.

Nevertheless, this study has several limitations. Transcriptomic analyses were performed using a limited number of biological donors, and conclusions are therefore framed to describe consistent transcriptional responses to substrate and flow conditions rather than inter-individual variability. Bulk RNA sequencing captures averaged gene expression across heterogeneous epithelial populations and does not resolve cell-type–specific contributions. In addition, functional assays, such as direct measurements of enzymatic activity or epithelial barrier function, were not performed, and findings are interpreted at the level of gene expression rather than functional output. Organoids at passages five to six were used, and phenotypic drift at higher passages cannot be excluded. Finally, physicochemical characterization of the dSIS-MA hydrogels was not repeated in this study, as the same material and fabrication protocols were used as previously reported (Almalla et al., 2024).

Together, by integrating dSIS-MA hydrogels with controlled fluidic shear stress, we established a biomimetic *in vitro* model capturing key transcriptional features of the human ileal epithelium, including epithelial differentiation–associated, metabolic, and immune-related transcriptional programs. This platform enhances barrier signaling, while shear stress further modulates epithelial responses, including CYP3A4, thereby helping to narrow the gap between conventional culture systems and *in vivo* intestinal physiology. Such biomimetic culture strategies, in line with recent advances demonstrating the regenerative potential of organoid–matrix combinations for intestinal graft engineering (Patel, 2018), hold strong potential for pharmaceutical research, disease modeling, and regenerative medicine.

## Data Availability

The data presented in the study are deposited in the GEO repository (https://www.ncbi.nlm.nih.gov/geo/), accession number GSE320151.
