# Supplementary material for: Transcriptomic profiling reveals substrate- and shear stress-dependent maturation of human small intestinal epithelial cells
Source: Front Pharmacol. 2026 May 1;17:1706106. doi: 10.3389/fphar.2026.1706106 (PMC13175875; doi:10.3389/fphar.2026.1706106)
Supplement: Supplementary file 1 [file Table1.pdf]

Supplementary Tables

Transcriptomic profiling reveals substrate- and shear stress-dependent maturation of human small intestinal epithelial cells

Nadra Alzain[1,2], Ahed Almalla[2], Ketaki Sandu[1,2], Lorenz Gerbeth[1], Jörn F. Ziegler[1,3], Catharina Gutwein[1], Patrick Weidner[1,4,5], Rainer Glauben[1], Marie Weinhart[2,6], Britta Siegmund[1,7]

Affiliations

[1] Charité - Universitätsmedizin Berlin, corporate member of Freie Universität Berlin and Humboldt-Universität zu Berlin, Department of Gastroenterology, Infectious Diseases and Rheumatology, Berlin, Germany

[2] Institute of Chemistry and Biochemistry, Freie Universität Berlin, Berlin, Germany

[3] Berlin Institute of Health at Charité – Universitätsmedizin Berlin, BIH Charité Junior Clinician Scientist Program, Berlin, Germany

[4] Berlin Institute for Medical Systems Biology, Max Delbrück Center for Molecular Medicine in the Helmholtz Association, Berlin, Germany

[5] Berlin Institute of Health at Charité – Universitätsmedizin Berlin

[6] Institute of Physical Chemistry and Electrochemistry, Leibniz Universität Hannover, Callinstr. 3A, 30167 Hannover, Germany

[7] Cluster of Excellence ImmunoPreCept, Charité – Universitätsmedizin Berlin, Berlin, Germany.

[*]*Corresponding author:*

Britta Siegmund

Charité - Universitätsmedizin Berlin, Medical Department, Division of Gastroenterology, Infectious Diseases and Rheumatology, Campus Benjamin Franklin

Hindenburgdamm 30, 12200 Berlin, Germany

phone: +49 30 450 614342, eMail: britta.siegmund@charite.de

Key words: epithelial cells, biomimetic, small intestine, organoids, shear stress, hydrogel, millifluidics

Supplementary Table 1. Primer Sequences.

Gene		Sequence 5' – 3'
CYP3A4	Forward	CCGAGTGGATTTCCTTCAGCTG
	Reverse	TGCTCGTGGTTTCATAGCCAGC
HPRT1	Forward	AGGGTGTTTATTCCTCATGGAC
	Reverse	CACAGAGGGCTACAATGTGAT
IL17RB	Forward	ATGTCGCTCGTGCTGCTAAG
	Reverse	AGCCACATTGAACGGTCGG
MAPK11	Forward	AAGCACGAGAACGTCATCGG
	Reverse	TCACCAAGTACACTTCGCTGA
MUC2	Forward	CAGAGGGCAGAACCCGAAAC
	Reverse	GAAGTTGTAGTCGCAGGGGC
NFKB1	Forward	AACAGAGAGGATTTCGTTTCCG
	Reverse	TTTGACCTGAGGGTAAGACTTCT
OCLN	Forward	ACAAGCGGTTTTATCCAGAGTC
	Reverse	GTCATCCACAGGCGAAGTTAAT
POLR2A	Forward	ACCTGATGGGCAAAAGAGTG
	Reverse	GCGCACTAGTTCTTGAAGTCTG
SPINK4	Forward	CAGTGGGTAATCGCCCTGG
	Reverse	CACAGATGGGCATTCTTGAGAAA
TBP	Forward	ACCCAGGGTGCCATGACT
	Reverse	CTGCTGCCTTTGTTGCTCTT
TGFB1	Forward	CCCAGCATCTGCAAAGCTC
	Reverse	GTCAATGTACAGCTGCCGCA
TNF	Forward	CTCAGCCTCTTCTCCTTC
	Reverse	AGAAGATGATCTGACTGCC
VEGFA	Forward	AGGGCAGAATCATCACGAAGT
	Reverse	AGGGTCTCGATTGGATGGCA
GAPDH	Forward	ACCCACTCCTCCACCTTTGAC
	Reverse	TGTTGCTGTAGCCAAATTCGTT

CDH1	Forward<br>Reverse	ATTTTTCCCTCGACACCCGAT<br>TCCCAGGCGTAGACCAAGA
TJP1	Forward<br>Reverse	CAACATACAGTGACGCTTCACA<br>CACTATTGACGTTTCCCCACTC
MKI67	Forward<br>Reverse	AAGAGAGTGTCTATCAGCCGAAGT<br>GTGGCCTGTACTAAATTGACTGTG
OLFM4	Forward<br>Reverse	AAATGCTCGAGAGTTGCGGA<br>CACAGCAATCGTGTTGGTGG
SI	Forward<br>Reverse	TTTGGCAGCCTTATCCAAGTC<br>ACAGCAGGTGTCTTAGTTGCT
ALPI	Forward<br>Reverse	CATCCAGAAGGTCGCCAAGA<br>TGCCATTCTTCTGCCCCTTT
CHGA	Forward<br>Reverse	CTACGCGCCTTGTCTCCTAC<br>GCTGCCTGTGTTCAGAGAAG
LYZ	Forward<br>Reverse	GCCAAATGGGAGAGTGGTTA<br>TAACTGCTCCTGGGGTTTTG
