# Supplementary material for: Transcriptomic profiling reveals substrate- and shear stress-dependent maturation of human small intestinal epithelial cells
Source: Front Pharmacol. 2026 May 1;17:1706106. doi: 10.3389/fphar.2026.1706106 (PMC13175875; doi:10.3389/fphar.2026.1706106)
Supplement: Supplementary file 2 [file Table2.pdf]

Supplementary Tables

Transcriptomic profiling reveals substrate- and shear stress-dependent maturation of human small intestinal epithelial cells

Nadra Alzain[1,2], Ahed Almalla[2], Ketaki Sandu[1,2], Lorenz Gerbeth[1], Jörn F. Ziegler[1,3], Catharina Gutwein[1], Patrick Weidner[1,4,5], Rainer Glauben[1], Marie Weinhart[2,6], Britta Siegmund[1,7]

Affiliations

[1] Charité - Universitätsmedizin Berlin, corporate member of Freie Universität Berlin and Humboldt-Universität zu Berlin, Department of Gastroenterology, Infectious Diseases and Rheumatology, Berlin, Germany

[2] Institute of Chemistry and Biochemistry, Freie Universität Berlin, Berlin, Germany

[3] Berlin Institute of Health at Charité – Universitätsmedizin Berlin, BIH Charité Junior Clinician Scientist Program, Berlin, Germany

[4] Berlin Institute for Medical Systems Biology, Max Delbrück Center for Molecular Medicine in the Helmholtz Association, Berlin, Germany

[5] Berlin Institute of Health at Charité – Universitätsmedizin Berlin

[6] Institute of Physical Chemistry and Electrochemistry, Leibniz Universität Hannover, Callinstr. 3A, 30167 Hannover, Germany

[7] Cluster of Excellence ImmunoPreCept, Charité – Universitätsmedizin Berlin, Berlin, Germany.

[*]Corresponding author:

Britta Siegmund

Charité - Universitätsmedizin Berlin, Medical Department, Division of Gastroenterology, Infectious Diseases and Rheumatology, Campus Benjamin Franklin

Hindenburgdamm 30, 12200 Berlin, Germany

phone: +49 30 450 614342, eMail: britta.siegmund@charite.de

Key words: epithelial cells, biomimetic, small intestine, organoids, shear stress, hydrogel, millifluidics

Supplementary Table 2. Reagents

Reagent	Supplier	Location
D-sorbitol	Carl Roth	Karlsruhe, Germany
Sucrose	Thermo Fisher Scientific	Waltham, MA, USA
Ethylenediaminetetraacetic acid (EDTA)	Merck	Darmstadt, Germany
Dithiothreitol (DTT)	Thermo Fisher Scientific	Waltham, MA, USA
Phosphate-buffered saline (PBS)	Thermo Fisher Scientific	Waltham, MA, USA
Ciprofloxacin	Fresenius Kabi	Bad Homburg, Germany
Gentamycin	Merck	Darmstadt, Germany
Fluconazole	B. Braun SE	Melsungen, Germany
Primocin	Invivogen	San Diego, CA, USA
Advanced DMEM/F-12	Thermo Fisher Scientific	Waltham, MA, USA
Penicillin/Streptomycin (Pen/Strep)	Biochrom AG	Berlin, Germany
Glutamine	Merck	Darmstadt, Germany
HEPES buffer	Gibco	Waltham, MA, USA
TrypLE Express Enzyme	Thermo Fisher Scientific	Waltham, MA, USA
Matrigel growth factor-reduced	Corning	Corning, NY, USA
L-WRN cells (CRL-3276)	ATCC	Manassas, VA, USA
Recombinant human EGF	Peprotech	Cranbury, NJ, USA
SB 202190	Merck	Darmstadt, Germany
A83-01	Merck	Darmstadt, Germany
[Leu15]-Gastrin I human	Merck	Darmstadt, Germany
Nicotinamide	Merck	Darmstadt, Germany
Y-27632 (Rock inhibitor)	Abmole	Houston, TX, USA
QIAzol lysis reagent	Qiagen	Hilden, Germany
Direct-zol RNA Microprep kit (R2062)	Zymo Research	Irvine, CA, USA

Agilent 4200 TapeStation System	Agilent Technologies	Santa Clara, CA, USA
High-sensitivity RNA ScreenTape Assay	Agilent Technologies	Santa Clara, CA, USA
DNase I amplification grade	Thermo Fisher Scientific	Waltham, MA, USA
dNTP ROTI Mix PCR	Carl Roth	Karlsruhe, Germany
RNaseOUT recombinant ribonuclease inhibitor	Thermo Fisher Scientific	Waltham, MA, USA
M-MLV Reverse Transcriptase	Thermo Fisher Scientific	Waltham, MA, USA
Oligo(dT)12-18 Primer	Thermo Fisher Scientific	Waltham, MA, USA
PowerUp SYBR Green Master Mix	Thermo Fisher Scientific	Waltham, MA, USA
Primers	TIB MolBiol	Berlin, Germany
QuantStudio 5 Real-Time PCR System	Applied Biosystems	Waltham, MA, USA
